# Niclosamide Ethanolamine Salt Alleviates Idiopathic Pulmonary Fibrosis by Modulating the PI3K-mTORC1 Pathway

**DOI:** 10.3390/cells11030346

**Published:** 2022-01-20

**Authors:** Xiaolin Pei, Fangxu Zheng, Yin Li, Zhoujun Lin, Xiao Han, Ya Feng, Zhenhuan Tian, Dunqiang Ren, Ke Cao, Chenggang Li

**Affiliations:** 1 State Key Laboratory of Medicinal Chemical Biology and College of Pharmacy, Nankai University, Tianjin 300350, China; peixiaolin@mail.nankai.edu.cn (X.P.); zhengfangxu2021@163.com (F.Z.); 1120200593@mail.nankai.edu.cn (Y.L.); linzhoujun@mail.nankai.edu.cn (Z.L.); Hanxiao@mail.nankai.edu.cn (X.H.); fengya@mail.nankai.edu.cn (Y.F.); 2 Department of Thoracic Surgery, Peking Union Medical College Hospital, Peking Union Medical College, Beijing 100730, China; tianzhenhuan@pumch.cn; 3 Department of Respiratory and Critical Care Medicine, The Affiliated Hospital of Qingdao University, Qingdao 266000, China; Rdq1982@163.com; 4 Department of Pathophysiology, Jinzhou Medical University, Jinzhou 121001, China

**Keywords:** niclosamide ethanolamine salt, idiopathic pulmonary fibrosis, epithelial–mesenchymal transition, cell proliferation, mTOR pathway

## Abstract

Idiopathic pulmonary fibrosis (IPF) is an interstitial pneumonia characterized by chronic progressive fibrosis, ultimately leading to respiratory failure and early mortality. Although not fully explored, the major causative factors in IPF pathogenesis are dysregulated fibroblast proliferation and excessive accumulation of extracellular matrix (ECM) deposited by myofibroblasts differentiated from pulmonary fibroblasts. More signalling pathways, including the PI3K-Akt-mTOR and autophagy pathways, are involved in IPF pathogenesis. Niclosamide ethanolamine salt (NEN) is a highly effective multitarget small-molecule inhibitor reported in antitumor studies. Here, we reported that in an IPF animal model treated with NEN for 14 days, attractive relief of pulmonary function and hydroxyproline content were observed. To further explore, the therapeutic effect of NEN in IPF and pathological changes in bleomycin-challenged mouse lung sections were assessed. Additionally, the effects of NEN on abnormal proliferation and ECM production in IPF cell models established with TGF-β1-stimulated A549 cells or DHLF-IPF cells were studied. In nonclinical studies, NEN ameliorated lung function and histopathological changes in bleomycin-challenged mice, and the lung hydroxyproline content was significantly diminished with NEN treatment. In vitro, NEN inhibited PI3K-mTORC1 signalling and arrested the cell cycle to prevent uncontrolled fibroblast proliferation. Additionally, NEN inhibited TGF-β1-induced epithelial–mesenchymal transition (EMT) and ECM accumulation via the mTORC1-4EBP1 axis. Furthermore, NEN-activated noncanonical autophagy resensitized fibroblasts to apoptosis. The above findings demonstrated the potential antifibrotic effect of NEN mediated via modulation of the PI3K-mTORC1 and autophagy pathways. These data provide strong evidence for a therapeutic role for NEN in IPF.

## 1. Introduction

Idiopathic pulmonary fibrosis (IPF) is characterized by progressive dyspnea and deterioration of lung function and is diagnosed as a serious lung disease with extracellular matrix (ECM) deposition and lung scarring; IPF has a high incidence and lacks effective therapeutic medicine [1].

Although the precise molecular and cellular mechanisms still require full elucidation, there is substantial evidence supporting the concept that a profibrotic environment orchestrated by underlying factors such as genetic predisposition, chronic injury and aging, oxidative stress [2,3], and impaired regenerative responses may account for disease development and persistence [4]. By producing growth factors, cytokines, chemokines, ECM proteins, and proteases, myofibroblasts play a pivotal role in fibrosis. Moreover, apoptosis, senescence, epithelial–mesenchymal transition, endothelial–mesenchymal transition, and epithelial cell migration have been shown to play a key role in IPF-associated tissue remodelling [5]. There are two complicated pathogenic processes in IPF. First, alveolar epithelial cells proliferate abnormally after recurrent damage, and the production of a variety of cytokines, especially transforming growth factor (TGF)-β1, aggravates epithelial cell damage, leading to chronic inflammation and epithelial–mesenchymal transition (EMT) [6]. Another causative factor is that hyperactive lung fibroblasts transform into myofibroblasts to repair damaged tissues, resulting in fibrotic foci [7]. The main features of IPF are the occurrence of EMT and (myo)fibroblast proliferation, which induce cytoskeletal changes, ECM composition alterations, and abnormal cell hyperplasia.

Mammalian target of rapamycin (mTOR) is composed of two protein complexes that have different functions, mTOR complex 1 (mTORC1) and mTOR complex 2 (mTORC2). In general, mTORC1 controls cell growth by regulating mRNA translation via phosphorylation of ribosomal S6 kinase (S6K) and 4E binding protein 1 (4E-BP1) [8]. In contrast, mTORC2 regulates cell proliferation, survival, apoptosis, growth, and the actin cytoskeleton by activating Akt, protein kinase C-alpha (PKC-alpha) and serum glucocorticoid-induced protein kinase-1 (SGK1) [9]. In response to energy deficiency, autophagy is activated by mTORC1 to degrade cellular components to maintain the necessary activities and survival of cells. mTORC1 promotes the large-scale, high-speed synthesis of EMT and ECM proteins to accelerate the progression of IPF [10]. Studies have shown that EMT and myofibroblast proliferation induced by excessive TGF-β1 related to the regulation of mTORC1 upstream molecules [11]. PI3K/Akt is one of the key upstream regulators of mTOR and plays an important role in regulating mTOR, and it has been proved that PI3K/mTOR pathway is activated in IPF. New small molecule inhibitors that target PI3K/mTOR pathway components have recently entered clinical trials [12]. According to a previous theory, PI3K/mTORC1 might be a potential therapeutic target in IPF.

Pirfenidone and nintedanib, which have been approved by the FDA for the treatment of IPF, improve the quality of life but not the survival rate of IPF patients [13]. New agent research and development requires many technological, financial and temporal costs. If an existing clinical medicine can be shown to treat IPF, it will be a very meaningful discovery.

Niclosamide (5-chloro-salicyl-(2-chloro-4-nitro) anilide) is a salicylamide derivative originally used as a molluscicide, and the FDA has approved its clinical use for the treatment of human and animal tapeworm infections, in which it interferes with mitochondrial metabolism [14] and oxidative phosphorylation uncoupling [15]. Niclosamide ethanolamine salt (NEN) is the salt form of niclosamide. This form has increased water solubility but the same pharmacological activity.

In recent years, studies have revealed the antitumor activity of NEN, mainly focusing on colorectal cancer [16], breast cancer [17], and leukemia [18]. NEN not only inhibits mitochondrial uncoupling but also affects a variety of signalling pathways, including the Wnt/β-catenin, mTORC1, STAT3, and NF-κb pathways [19]. In addition, NEN has been used to improve glucose and lipid metabolism disorders by inhibiting mitochondrial metabolism in the treatment of liver fibrosis [20]. Despite the status of NEN as a highly effective multitarget small-molecule inhibitor, however, there is little research on its effect on IPF.

In the present study, we investigated the antifibrotic effect and underlying therapeutic mechanism of NEN in IPF. Based on the data presented, we found that NEN inhibited EMT and cell proliferation by modulating PI3K-mTORC1 and the autophagy pathway to treat IPF.

## 2. Materials and Methods

### 2.1. BLM-Induced IPF Mouse Model

Male C57BL-6J mice (body weight: 20 ± 2 g) were purchased from Charles River (Beijing, China) and housed at 22–24 °C with a relative humidity of 50–60% and a 12:12 h light-dark cycle. An IPF mouse model was established using bleomycin (BLM) (Sigma–Aldrich, St. Louis, MI, USA) or 0.9% NaCl solution (blank group) at 2.5 mg/kg body weight administered with a mouse liquid lung administration nebulizer. NEN (20 mg/kg) was intragastrically administered daily for 14 days, beginning 7 days post-BLM administration, and the blank group was intragastrically administered 0.5% CMC-Na. Body weight was monitored and recorded daily. Mice were sacrificed after treatment with NEN or CMC-Na for 14 days. Pulmonary function was measured using an Anires2005 system (Beijing, China) before sacrifice.

### 2.2. Cell Culture and Reagents

The human pulmonary epithelial A549 cell line was obtained from Fenghui Biological Technology Co., Ltd. (Hunan, China). Diseased human lung fibroblasts and idiopathic pulmonary fibrosis (DHLF-IPF cells) were supplied by Dr. Ke Cao (Jinzhou Medical University) and Dr. Dunqiang Ren (The Affiliated Hospital of Qingdao University) [21]. A549 cells were cultured in Ham’s F-12K medium (Gibco, Grand Island, NY, USA), and DHLF-IPF cells were cultured in DMEM (Gibco, Grand Island, NY, USA); both media were supplemented with 1% penicillin–streptomycin (Gibco, Grand Island, NY, USA) and 10% FBS (Gibco, Grand Island, NY, USA). Cultured cells were maintained at 37 °C in a 5% CO_2_ incubator. To induce EMT, A549 cells were cultured in 0.1% FBS medium for 6 h before 10 ng/mL TGF-β1 (PeproTech, Rocky Hill, NJ, USA) was added to induce fibrotic events. Cells were grown to 70% confluence before inclusion in experiments. A NEN stock solution at a concentration of 1 mM was used (solvent: DMSO), stored at −20 °C and diluted to the indicated working concentrations before use.

### 2.3. Cell Viability and Cell Death Assays

Cells were seeded in a 96-well plate at a density of 8000 cells per well and cultured overnight. A549 cells were treated with TGF-β1 (10 ng/mL) to induce EMT for 24 h. TGF-β1-induced A549 cells and DHLF-IPF cells were treated with different concentrations of NEN for 24 h or 48 h. Cell Counting Kit-8 (CCK-8; MeilunBio, Dalian, China) reagent was added to the cells (10 μL per well) and incubated for 1.5 h to measure cell viability and calculate the half maximal inhibitory concentration (IC_50_). Propidium iodide (PI; Solarbio Life Science, Beijing, China) and crystal violet (CV; Solarbio Life Science, Beijing, China) staining were used to evaluate cell death. Cells were stained with 0.5 mg/mL PI for 45 min, and the fluorescence was measured. Then, the cells were fixed with formalin, 100 μL of crystal violet was added to each well, and the absorbance was detected with a microplate reader. Cell death is presented as the PI fluorescence/CV absorbance ratio.

### 2.4. Wound-Healing Assays

A549 cells and DHLF-IPF cells were seeded in a 6-well plate at a density of 10^5^ cells per well. A549 cells were cultured with or without TGF-β1 (10 ng/mL) for another 24 h. Two hundred-microliter micropipette tips were used to make a vertical scratch in the well center. TGF-β1-induced A549 cells were cultured in the absence or presence of NEN (0.5 μM) with TGF-β1 (10 ng/mL) in 0.1% FBS culture medium. DHLF-IPF cells were treated with NEN (1 μM) in 1% FBS culture medium. At different time points, images of the scratch width in each well were acquired by light microscopy imaging and analysed using ImageJ software.

### 2.5. Western Blot Analysis

Cells and mouse lung tissues were lysed with RIPA buffer containing protease inhibitors (Solarbio Life Science, Beijing, China) and phosphatase inhibitors (Solarbio Life Science, Beijing, China) and centrifuged at 4 °C to obtain the supernatant. The concentration of total protein was detected with a BCA kit (Thermo Fisher Scientific, Waltham, MA, USA). Ten micrograms of total protein were added to each lane of SDS-polyacrylamide gels, and then the separated protein samples were transferred to PVDF membranes (Immobilon^®^-P, Darmstadt, Germany) and incubated with primary antibodies overnight at 4 °C. After incubation with an HRP-labeled secondary antibody for 1 h at room temperature, an ECL Chemiluminescence Detection Kit (Thermo Fisher Scientific, Waltham, MA, USA) was used for visualization and imaging with a Tanon 5200 automatic chemiluminescence fluorescence image analysis system (Shanghai, China). The results were analysed with ImageJ software.

Anti-phospho-mTOR (S2448), anti-phospho-AKT (S473), and anti-COL1A1 (Col-I) antibodies were obtained from Santa Cruz Biotechnology (Santa Cruz, CA, USA). An anti-fibronectin (FN) antibody was purchased from BD Biosciences (Franklin Lake, NJ, USA). Antibodies against α-smooth muscle actin (α-SMA), E-cadherin (E-cad), vimentin (VIM), phospho-S6 ribosomal protein (S235/236), S6 ribosomal protein, phospho-4E-BP1 (T70), 4E-BP1, LC3B, Akt and mTOR were purchased from Cell Signaling Technology (Danvers, MA, USA). An anti-SQSTM1/p62 antibody was purchased from Bioworld Technology (Nanjing, China), and an anti-GAPDH antibody was purchased from Abcam (Cambridge, UK).

### 2.6. RNA Isolation and Quantitative Real-Time PCR

Total RNA was isolated from cells and lung tissues with TRIzol Reagent (Invitrogen, Grand Island, NY, USA). Total RNA concentrations were determined using an ultraviolet spectrophotometer. cDNA (reverse transcription) was generated using the Prime Script™ RT Master Mix Kit (TaKaRa, Dalian, Liaoning, China) according to the manufacturer’s instructions. Real-time quantitative PCR (qPCR) was performed using a SuperReal PreMix Plus (SYBR Green) (TIANGEN, Beijing, China) and a Bio–Rad CFX Maestro system. Target mRNA expression was normalized to *GAPDH* expression. Primers were synthesized by AuGCT DNA-SYN Biotechnology (Beijing, China).

Human primer sequences:

*GAPDH* forward 5′ TCCAAAATCAAGTGGGGC 3′ and

*GAPDH* reverse 5′ ACTACTAGAACTCCGACA 3′.

*COL1A1* forward 5′ GAGGGCCAAGACGAAGACATC 3′ and

*COL1A1* reverse 5′ CAGATCACGTCATCGCACAAC 3′.

*ACTA2* forward 5′ GTGTTGCCCCTGAAGAGCAT 3′ and

*ACTA2* reverse 5′ GCTGGGACATTGAAAGTCTCA 3′.

*CDH1* forward 5′ CGAGAGCTACACGTTCACGG3′ and

*CDH1* reverse 5′ GGGTGTCGAGGGAAAAATAGG 3′.

*CDH2* forward 5′ AGCCAACCTTAACTGAGGAGT 3′ and

*CDH2* reverse 5′ GGCAAGTTGATTGGAGGGATG 3′.

*VIM* forward 5′ AGTCCACTGAGTACCGGAGAC 3′ and

*VIM* reverse 5′ CATTTCACGCATCTGGCGTTC 3′.

*FN1* forward 5′ CGGTGGCTGTCAGTCAAAG 3′ and

*FN1* reverse 5′ AAACCTCGGCTTCCTCCATAA 3′.

*ATG5* forward 5′ AAAGATGTGCTTCGAGATGTGT 3′ and

*ATG5* reverse 5′ CACTTTGTCAGTTACCAACGTCA 3′.

*ATG7* forward 5′ CAGTTTGCCCCTTTTAGTAGTGC 3′ and

*ATG7* reverse 5′ CCAGCCGATACTCGTTCAGC 3′.

*ATG12* forward 5′ CTGCTGGCGACACCAAGAAA 3′ and

*ATG12* reverse 5′ CGTGTTCGCTCTACTGCCC 3′.

Mouse primer sequences:

*Gapdh* forward 5′ CATCACTGCCACCCAGAAGACTG 3′ and

*Gapdh* reverse 5′ ATGCCAGTGAGCTTCCCGTTCAG 3′.

*Sqstm1* forward 5′ AGGATGGGGACTTGGTTGC 3′ and

*Sqstm1* reverse 5′ TCACAGATCACATTGGGGTGC 3′.

*Atg7* forward 5′ GTTCGCCCCCTTTAATAGTGC 3′ and

*Atg7* reverse 5′ TGAACTCCAACGTCAAGCGG 3′.

*Col1a1* forward 5′ GCTCCTCTTAGGGGCCACT 3′ and

*Col1a1* reverse 5′ ATTGGGGACCCTTAGGCCAT 3′.

*Acta2* forward 5′ GGCACCACTGAACCCTAAGG 3′ and

*Acta2* reverse 5′ ACAATACCAGTTGTACGTCCAGA 3′.

*Cdh1* forward 5′ TCGGAAGACTCCCGATTCAAA 3′ and

*Cdh1* reverse 5′ CGGACGAGGAAACTGGTCTC 3′.

*Vim* forward 5′ CCACACGCACCTACAGTCT 3′ and

*Vim* reverse 5′ CCGAGGACCGGGTCACATA 3′.

*Fn1* forward 5′ TCAAGTGTGATCCCCATGAAG 3′ and

*Fn1* reverse 5′ CAGGTCTACGGCAGTTGTCA 3′.

### 2.7. Immunofluorescence Confocal Microscopy

Cells were incubated in 12-well plates containing glass plates and cultured overnight. After EMT induction and NEN treatment, 4% paraformaldehyde (PFA) was added at room temperature for 20–30 min. Endogenous peroxidase activity was blocked with 3% hydrogen peroxide. The cells were treated with 0.5% Triton for 5 min. Then, the cells were incubated with 3% goat serum (Solarbio Life Science, Beijing, China) to block nonspecific binding at room temperature for 1 h. Then, a primary antibody was added and incubated at 4 °C overnight, and an appropriate HRP-labeled secondary antibody was added and incubated at room temperature for 1 h. DAPI (0.5 μg/mL, Solarbio Life Science, Beijing, China) was used to stain nuclei for 15 min, and 30% glycerin was added to seal the glass slides with another slide. The slides were observed and imaged with a confocal microscope.

### 2.8. Cell Cycle Detection

A549 cells were incubated in 6-well plates and cultured overnight. After EMT induction and NEN treatment, the cells were treated according to the JC-1 kit instructions (MultiSciences, Hangzhou, China) and detected with flow cytometry.

### 2.9. Histology and Immunohistochemistry

The left lung tissue of mice was fixed with 4% PFA for 2 days, embedded in paraffin and sectioned. After deparaffinization and gradient alcohol hydration, paraffin tissue sections (5 μM) were stained with the Hematoxylin-Eosin Staining Kit or Masson Trichrome Stain Kit (Solarbio Life Science, Beijing, China). For immunohistochemistry, a 3%-hydrogen-peroxide solution was added dropwise to tissue sections to quench endogenous peroxidase activity. The sections were then immersed in 0.01 M citrate (Solarbio Life Science, Beijing, China) buffer, and antigen retrieval was performed by the microwave method. Normal goat serum (10%) was added, and the sections were incubated for 1 h at room temperature to block nonspecific binding. The blocking solution was removed, a primary antibody was added dropwise, and the sections were incubated overnight at 4 °C. A secondary antibody working solution was added dropwise and incubated at room temperature for 1 h. A freshly prepared DAB working solution (Solarbio Life Science, Beijing, China) was dropped onto slides, and the degree of staining was monitored under a microscope. Finally, the sections were counterstained with hematoxylin for 2 min. The stained tissue sections were observed under a microscope (Leica, Wetzlar, Germany).

### 2.10. Hydroxyproline Assay

The right lung was accurately weighed according to the instructions of a hydroxyproline test kit (Nanjing Jiancheng Bioengineering Institute, Nanjing, China). The results are expressed as µg of hydroxyproline/mg of protein.

### 2.11. Gene Expression Array Reanalysis

Reanalysis of previously published expression array data (GEO accession number: GSE68239) deposited by Wilhelm J was performed using the online tool www.aclbi.com (accessed on 1 November 2021). The transcript levels of fibrotic genes (*VIM*, *ACTA2*, *FN1,* and *COL1A1*) and mTOR pathway genes (*ATG5*, *EIF4EBP1*, *MAP1LC3A,* and *RPS6*) in lung tissues were compared between 10 healthy donor tissue samples and 10 IPF patient samples. Correlation analysis was performed using the Pearson correlation coefficient, and correlograms were analysed with R v4.0.3.

### 2.12. IPF Patient Lung Tissue

IPF patient lung tissues were obtained from the pathological biopsy with IPF and contributed by Dr. Zhenhuan Tian (Peking Union Medical College Hospital). Control lung samples were obtained during surgical resection in patients with lung cancer as macroscopically uninvolved normal tissue.

### 2.13. Statistical Analysis

All data are presented as the mean ± SEM of at least three independent experiments (*n* ≥ 3). Student’s t test was used to compare two groups, and two-way ANOVA was used for multiple-group comparisons. Statistical significance was defined as *p* < 0.05. Graph production and statistical analysis were performed using GraphPad Prism (version 8.3.0).

## 3. Results

### 3.1. NEN Attenuated IPF in BLM-Induced Mice

**Figure 1:**
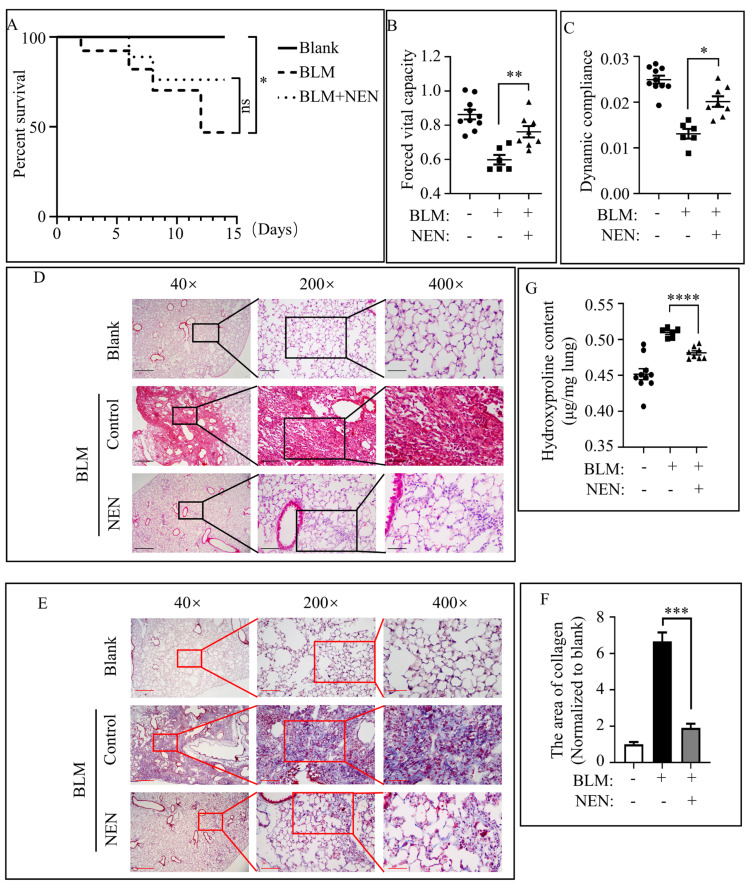
NEN attenuated IPF in BLM-induced mice. To evaluate the therapeutic effect of NEN in a preclinical model of pulmonary fibrosis, C57BL-6J mice were challenged with bleomycin (BLM) or a 0.9% NaCl solution as a vehicle control (blank) at 2.5 mg/kg body weight; the agents were administered with a liquid lung administration nebulizer. Beginning on day 7 post drug (vehicle) delivery, BLM-challenged mice received NEN (20 mg/kg) or vehicle treatment (i.g.) daily for 2 weeks. (**A**) The survival rate of each group of mice was counted (*n =* 10 in the blank group, *n =* 6 in the BLM group and *n =* 8 in the NEN group). Pulmonary function was evaluated by assessing parameters including (**B**) forced vital capacity (FVC) and (**C**) dynamic compliance (Cdyn) to compare the different treatments. Lung sections were stained with H and E (**D**) or Masson trichrome (**E**) to assess collagen accumulation (representative image, magnification 40×, bar = 500 µm, 200×, bar = 100 µm, 400×, bar = 50 µm), and Masson trichrome staining (magnification 40×) was quantified (**F**) by ImageJ software with comparison to the blank group. (**G**) The hydroxyproline content in lung tissues among the different groups was analysed and quantified. * *p* < 0.05, ** *p* < 0.01, *** *p* < 0.001, **** p < 0.0001.

To evaluate the potential antifibrotic effect of NEN in vivo, BLM was used to establish a preclinical IPF model (described in the Methods section). Body weight was monitored daily after intratracheal administration of BLM or vehicle. BLM mice treated with or without NEN showed limited differences, but their body weights were lower than those of blank mice (Supplementary Figure S1A). However, NEN improved the survival rate in bleomycin-induced pulmonary fibrosis mice (Figure 1A). Mice were sacrificed at the endpoint after NEN treatment for 14 days, and pulmonary functions, including forced vital capacity (FVC) and dynamic compliance (Cdyn), were significantly enhanced in the NEN (20 mg/kg) group compared with the BLM only group, which showed a decline in function, indicating that NEN treatment attractively relieved respiratory system dysfunction in the preclinical model (Figure 1B,C). In addition, BLM mice exhibited abundant accumulation of hydroxyproline, which indicated excess deposition of collagen in lung tissue. However, after NEN (20 mg/kg) treatment, fibrotic regions were reduced significantly with fewer inflammatory cells and decreased hydroxyproline content (Figure 1G).

Pathological changes in lung sections shown by hematoxylin and eosin (H and E) staining revealed that the normal alveolar structure in the BLM group was blurred or disappeared, along with an incomplete alveolar shape and scattered fibrotic foci. Cell nuclei were stained deep blue, indicating largely proliferative cells. Treatment with NEN (20 mg/kg) resulted in retention of a mostly intact alveolar structure and a markedly reduced fibrotic focus, with few proliferative cells (Figure 1D). Collagen deposition in lung sections, identified by Masson trichrome staining (Figure 1E) and quantified from the hydroxyproline content (Figure 1G), was strikingly increased in fibrotic foci in BLM mice compared with those in mice in the control group. Similarly, NEN (20 mg/kg) reduced collagen deposition (Figure 1E,G), and the area of collagen deposition was measured with ImageJ (Figure 1F).

### 3.2. NEN Inhibited Cell Viability and Migration in TGF-β1-Induced A549 Cells and DHLF-IPF Cells

NEN was shown to exhibit antifibrotic capacities in the in-vivo IPF model. To further explore the fibrosis-associated mechanism, we aimed to validate the findings in culture cell models. First, we investigated the effect of NEN on the EMT process, which contributes to collagen-producing fibroblasts in IPF and is therefore a hallmark of IPF. During the EMT process, downregulation of epithelial markers and upregulation of mesenchymal markers and ECM deposition are observed. In the process of EMT, TGF-β1 is considered to be a causative cytokine, crucial functions in the transdifferentiation of epithelial cells into fibroblasts and dysregulated proliferation [22].

**Figure 2:**
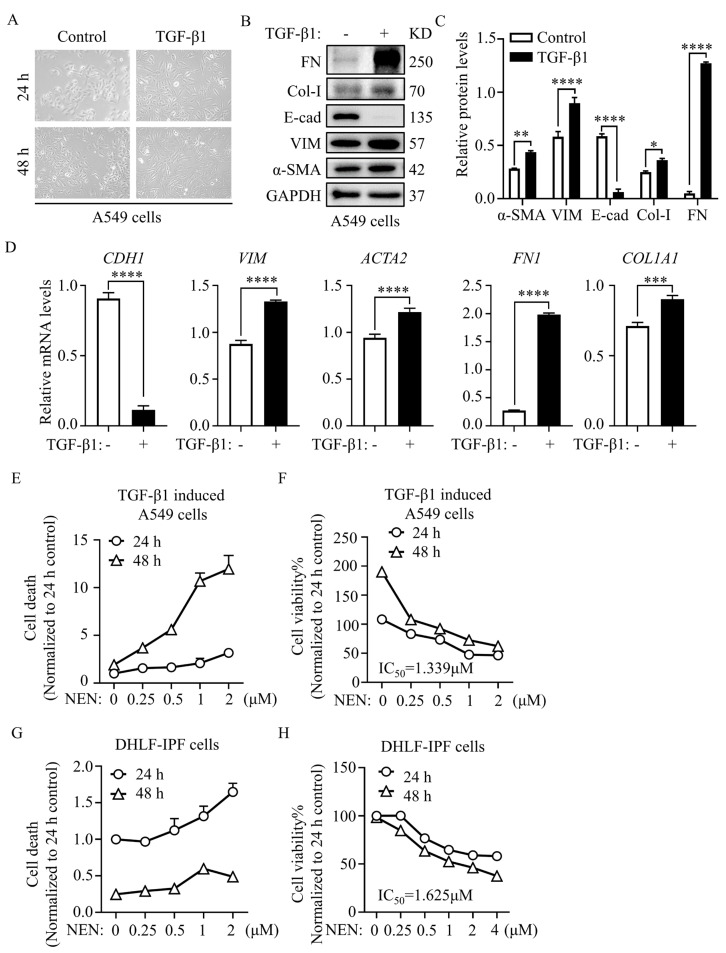
NEN inhibited cell viability in TGF-β1-induced A549 cells and DHLF-IPF cells. To establish a cellular model of EMT, A549 cells were pretreated with or without 10 ng/mL TGF-β1 for 24 or 48 h. (**A**) Changes in cell morphology were observed by light microscopy (magnification 200×). EMT-related gene and protein expression in A549 cells was measured after 10 ng/mL TGF-β1 or vehicle treatment for 24 h. (**B**) The protein levels of α-SMA, VIM, E-cad, Col-I, and FN were evaluated by an immunoblotting assay. (**C**) Densitometry was performed to analyse protein expression, and GAPDH was used as an internal reference. (**D**) The mRNA levels of *CDH1, VIM, ACTA2, FN1,* and *COL1A1* were detected by qPCR and normalized to the *GAPDH* level. TGF-β1-induced A549 cells and diseased human lung fibroblasts and idiopathic pulmonary fibrosis (DHLF-IPF cells) were treated with NEN at the indicated doses for 48 h. Cell death (**E**,**G**) and cell viability (**F**,**H**) were determined by a PI exclusion assay and CCK-8 staining assay, respectively. The half maximal inhibitory concentration (IC_50_) was calculated after 24 h of NEN treatment. * *p* < 0.05, ** *p* < 0.01, *** *p* < 0.001, **** *p* < 0.0001.

TGF-β1-induced A549 cells were used in our study as an in-vitro EMT model. Briefly, 10 ng/mL TGF-β1 was added to the culture medium of A549 cells, and morphological changes were observed at 24 h post-treatment (Figure 2A). EMT protein markers were evaluated and used for comparison with normal cultured A549 cells. EMT transition in TGF-β1-induced A549 cells was accompanied by a decrease in the epithelial marker E-cad and increases in the mesenchymal markers VIM and α-SMA, and the ECM markers FN and COL-I were also elevated dramatically (Figure 2B,C). Similarly, the transcript levels of the above genes, including *CDH1*, *VIM*, *ACTA2*, *FN1*, and *COL1A1*, were changed accordingly during TGF-β1-induced EMT progression (Figure 2D).

Next, we performed cell viability and cell death assays to optimize the dose of NEN for cultured cell treatment. TGF-β1-induced A549 cells and DHLF-IPF cells were treated with NEN at the indicated dose for 24 or 48 h. Cell death (Figure 2E,G) was determined by PI exclusion, and CCK-8 reagent was used to assess cell viability (Figure 2F,H). The IC_50_ values of NEN after 48 h of treatment were 1.339 μM for TGF-β1-induced A549 cells (Figure 2F) and 1.625 μM (Figure 2H) for DHLF-IPF cells. Both TGF-β1-induced A549 cells and DHLF-IPF cells showed dose- and time-dependent responses to NEN treatment. In a subsequent antifibrosis study, we used NEN at 0.5 μM for TGF-β1-induced A549 cells and at 1.0 μM for DHLF-IPF cells to avoid interference from cytocidal effects.

**Figure 3:**
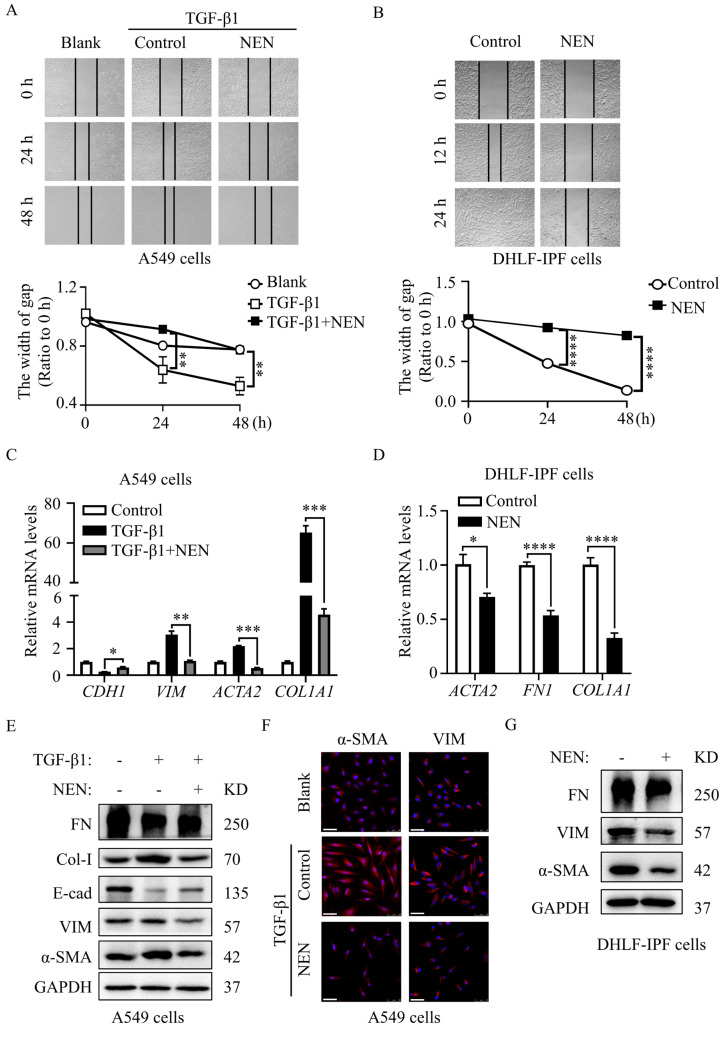
NEN treatment inhibited cell migration and reversed EMT in TGF-β1-induced A549 cells and DHLF-IPF cells. A wound-healing assay was performed to evaluate the effect of NEN on cell migration. The width of the scratch was photographed (upper panels) and quantified (lower panels) at 0, 24, and 48 h post-scratching of TGF-β1-induced A549 cells (**A**) or at 0, 12, and 24 h post-scratching of DHLF-IPF cells (**B**) (magnification 40×). The mRNA levels of *CDH1, VIM, ACTA2,* and *COL1A1* in (**C**) TGF-β1-induced A549 cells and (**D**) DHLF-IPF cells treated with or without NEN for 24 h were determined by qPCR. The results were normalized to the *GAPDH* level. Control or TGF-β1-induced A549 cells were treated with or without NEN (0.5 μM) for 24 h. The protein expressions of α-SMA, VIM, E-cad, Col-I, and FN were measured by an immunoblotting assay (**E**), and the cellular localization of α-SMA and VIM were determined by immunofluorescence staining (**F**) (magnification 400×, bar = 50 μm). (**G**) DHLF-IPF cells were exposed to NEN (1 μM) for 24 h, and the protein expressions of α-SMA, VIM, and FN were detected by an immunoblotting assay. * *p* < 0.05, ** *p* < 0.01, *** *p* < 0.001, **** *p* < 0.0001.

During the EMT process, the cytoskeletal structure is remodelled, and cell migration is enhanced. To test cell migration, we used a wound-healing assay to evaluate TGF-β1-induced A549 cells and DHLF-IPF cells in the presence or absence of NEN. Exogenous TGF-β1 (10 ng/mL)-treated A549 cells exhibited enhanced migration compared to regular A549 cells, while NEN (0.5 μM) treatment persistently affected the abnormal motility induced by TGF-β1 (Figure 3A). In DHLF-IPF cells, NEN significantly impeded wound-healing capacities (Figure 3B). In short, NEN disrupted abnormal cell migration during EMT processes.

### 3.3. NEN Reversed Elevations in EMT and ECM Markers in TGF-β1-Induced A549 and DHLF-IPF Cells

Given the limited wound-healing capacities seen in the context of NEN treatment, we investigated the regulatory effects of NEN on EMT and ECM-related biomarkers in TGF-β1-induced A549 cells and DHLF-IPF cells. NEN (0.5 μM) treatment for 24 h increased the mRNA levels of *CDH1* and evidently decreased *VIM*, *ACTA2,* and *COL1A1* expression (Figure 3C). Accordingly, the protein expression of VIM, α-SMA, FN, and Col-I was decreased, while E-cad expression was increased in response to NEN (0.5 μM) treatment in TGF-β1-induced A549 cells (Figure 3E and Supplementary Figure S1A). Furthermore, DHLF-IPF cells were treated with NEN (1 μM) for 24 h, and the mRNA levels of *ACTA2*, *FN1,* and *COL1A1* (Figure 3D), as well as the protein expression of α-SMA, VIM, and FN (Figure 3G and Supplementary Figure S1B), were all impeded. Furthermore, immunofluorescence staining for α-SMA and VIM in TGF-β1-induced A549 cells indicated that the elevated cellular α-SMA level and VIM deposition were blocked by NEN treatment, which suggested fibrotic deformation (Figure 3F). The reductions in the levels of mesenchymal proteins (α-SMA and VIM) and restoration of the epithelial marker E-cad, as well as the reduced ECM deposition (FN) and mobility change, suggested that the process of EMT was disrupted by NEN.

### 3.4. Antifibrotic Effect of NEN Mediated by PI3K/mTORC1 Pathway Blockade and Cell Cycle Arrest

**Figure 4:**
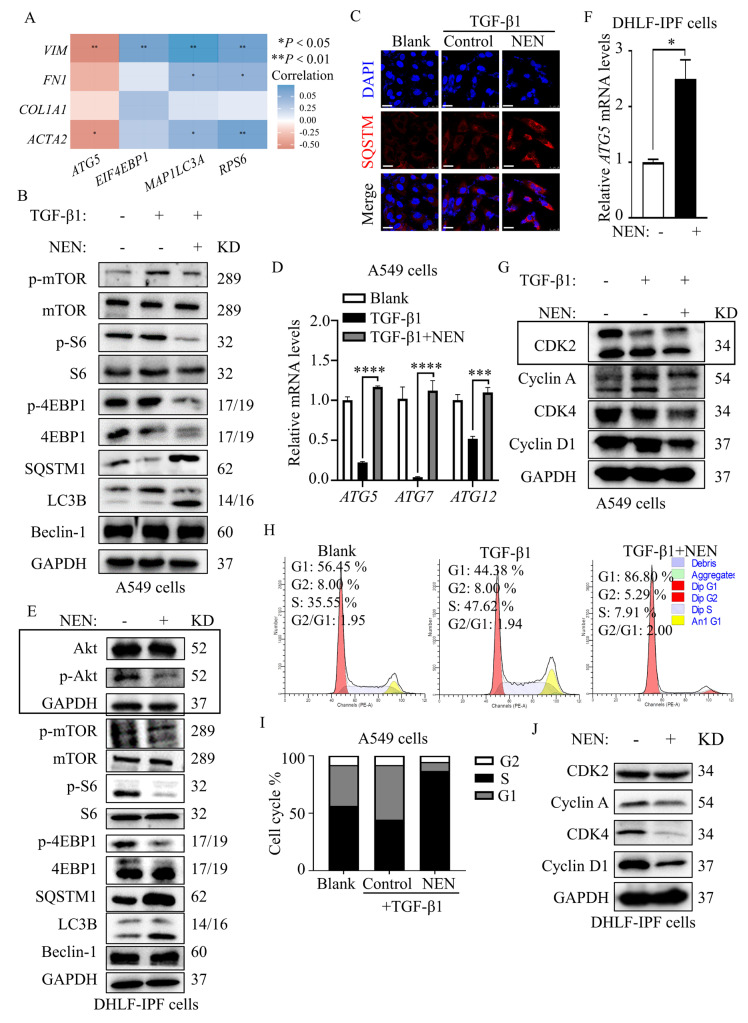
Antifibrotic effect of NEN mediated by mTORC1 pathway blockade and cell cycle arrest. (**A**) The correlogram was derived from reanalysis of a public dataset (GEO accession #: GSE68239). Blue represents a significant positive correlation (*p* < 0.05), red represents a significant negative correlation (*p* < 0.05), and white represents a nonsignificant correlation (*p* > 0.05). (**B**–**D**,**G**,**H**): Blank or TGF-β1-induced A549 cells were treated with or without NEN (0.5 μM) for 24 h. The expression patterns of proteins in (**B**) the mTORC1 pathway, including mTOR and phosphorylated mTOR (S2448), S6 and phosphorylated S6 (S235/236), 4EBP1 and phosphorylated 4EBP1 (T70), SQSTM1/p62, Beclin-1 and LC3B (I and II), and in (**G**)**,** the cell cycle pathway, including CDK2, Cyclin A, CDK4, and Cyclin D1, were detected by an immunoblotting assay; GAPDH was used as an internal reference. (**C**) Immunofluorescence staining was performed to detect the cellular localization of SQSTM1/p62, and nuclei were stained with DAPI. (**D**) The relative mRNA levels of ATGs (*ATG5, ATG7,* and *ATG12*) were detected by qPCR and normalized to the *GAPDH* level. (**H**) Cells were stained with JC-1 solution, and the cell cycle was analysed and quantified by flow cytometry. (**E**,**F**,**J**): DHLF-IPF cells were treated with a vehicle control or NEN (1 μM) for 24 h. Changes in the expression of proteins in (**E**) the PI3K/mTORC1 pathway, including Akt and phosphorylated Akt (S473), mTOR and phosphorylated mTOR (S2448), S6 and phosphorylated S6 (S235/236), 4EBP1 and phosphorylated 4EBP1 (T70), SQSTM1/p62, Beclin-1 and LC3B (I and II), and in (**I**)**,** the cell cycle pathway, including CDK2, Cyclin A, CDK4, and Cyclin D1, were detected by an immunoblotting assay. GAPDH was used as an internal reference. (**F**) Relative *ATG5* mRNA levels were detected by qPCR and normalized to the *GAPDH* level. * *p* < 0.05, ** *p* < 0.01, *** *p* < 0.001, **** *p* < 0.0001.

The FDA-approved drugs pirfenidone and nintedanib show limited curative effects, and more complex interactions have been reported; some of these interactions include the PI3K-mTOR-autophagy pathway, which integrates cell metabolism, proliferation, differentiation, and survival. Alterations in the PI3K-mTOR-autophagy pathway have been widely studied in cancer therapy, suggesting promising signalling modulation in IPF. To define the specific relationship between mTOR signalling and IPF, we examined differential gene expression and the correlations of the mTOR pathway with fibrotic markers using publicly available expression array datasets for lung sections collected from healthy donors and IPF patients (GEO accession number: GSE68239). The analysis showed that the transcript levels of genes in the mTOR pathway, including *EIF4EBP1, MAP1LC3A* and *RPS6,* were positively correlated with fibrotic lesions. Interestingly, the transcript levels of the autophagy gene *ATG5*, which is negatively regulated by mTOR, were negatively correlated with IPF (Figure 4A).

The PI3K-mTOR pathway is activated by cell-surface receptors, including tyrosine kinase receptors, and activated signalling regulates several downstream effectors, such as p70S6K-mediated protein synthesis and proliferation, which are sensitive to rapamycin treatment, and 4EBP1, the rapamycin-insensitive branch involved in the control of TGF-β1-stimulated collagen synthesis in fibroblasts. In addition, autophagy downstream of mTOR signalling is also involved in fibrotic conditions.

In TGF-β1-induced A549 cells, we observed constant activation of PI3K-mTOR signalling, indicated by increased levels of phosphorylated mTOR (S2448), phosphorylated S6 (S235/236), and phosphorylated 4EBP1 (T70) and decreased levels of SQSTM1/p62 and LC3B-I (Figure 4B and Supplementary Figure S2A). After NEN treatment, the phosphorylation of substrates, such as S6 and 4EBP1, by mTOR was blocked, and elevated LC3B II levels indicated that autophagy was restored. Beclin-1, a protein involved in the early stages of phagophore formation, remained intact with or without NEN treatment, suggesting that Beclin-1-independent noncanonical autophagy is involved (Figure 4B). Furthermore, accumulation of cellular SQSTM (Figure 4C) and restored expression of autophagy initiation genes, including *ATG5*, *ATG7,* and *ATG12,* were observed in TGF-β1-induced A549 cells treated with NEN (Figure 4D). Compared with the effective PI3K/Akt inhibitor wortmannin or the mTORC1-specific inhibitor rapamycin, NEN significantly blocked the phosphorylation of Akt and mTOR and restored LC3B II expression in TGF-β1-induced A549 cells (Supplementary Figure S2B,C). Unlike 4EBP1, p70S6 was sensitive to rapamycin at 20 nM, but a feedback loop led to Akt activation. In addition, rapamycin-treated cells maintained high levels of EMT-related proteins such as VIM, Col-I, and FN (Supplementary Figure S2D). Similarly, in DHLF-IPF cells, NEN inhibited the phosphorylation of Akt (S473), mTOR (S2448), S6 (S235/236) and 4E-BP1 (S65) and induced SQSTM1/p62 and LC3B-II accumulation identical to that observed in TGF-β1-induced A549 cells treated with NEN (Figure 4E and Supplementary Figure S2E). On the other hand, *ATG5* gene expression was undoubtedly restored by NEN (Figure 4F). The results for both TGF-β1-induced A549 cells and DHLF-IPF cells validated that NEN stabilized both rapamycin-sensitive and rapamycin-independent PI3K-mTOR signalling and restored Beclin-1-independent autophagy, which might contribute to NEN-mediated antiproliferative effects and decrease in the ECM.

Surprisingly, we also found that NEN blocked the cell cycle in the G1 phase (Figure 4H) by negatively regulating cyclin/CDK complexes (cyclin A/CDK2 and cyclin D1/CDK4) in both TGF-β1-induced A549 cells (Figure 4G and Supplementary Figure S2F) and DHLF-IPF cells (Figure 4I and Supplementary Figure S2G). Indeed, cell cycle proteins are frequently hyperactive during IPF progression, and blocking cell cycle progression through cell cycle protein inhibition can lead to cell proliferation arrest. Combined with its modulatory effect on the PI3K-mTOR-autophagy pathway, NEN impacted abnormal proliferation in fibrosis via dual inhibition of the cell cycle and S6K signalling and limited ECM deposition via 4EBP1 inhibition, which affected IPF cell growth and proliferation.

**Figure 5:**
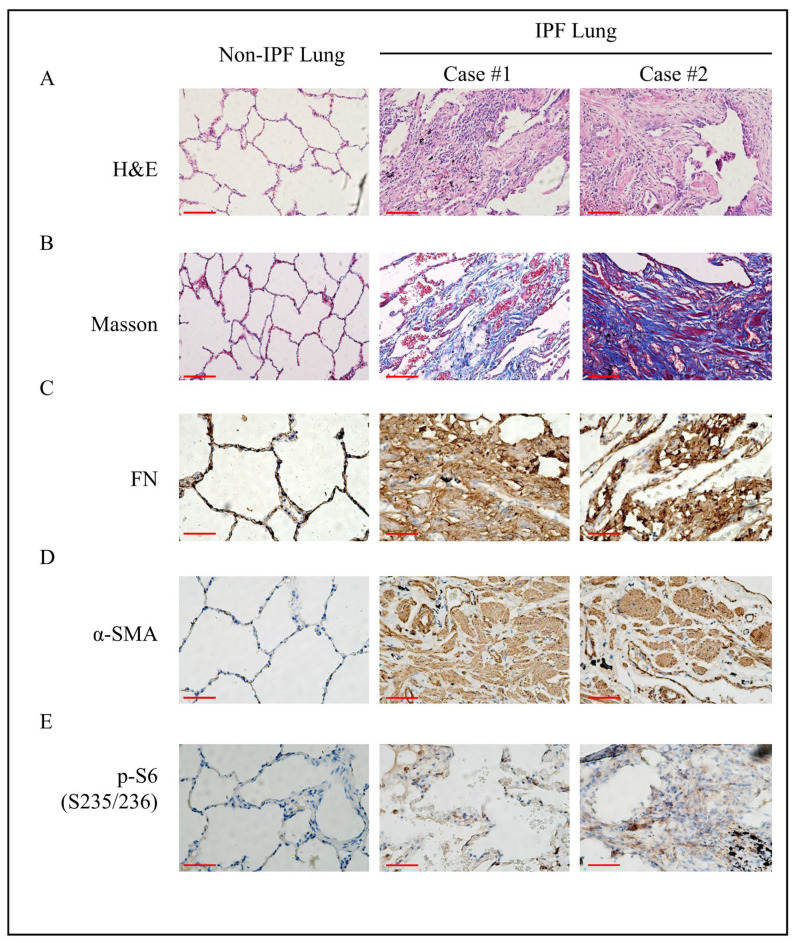
Phosphorylated S6 accumulated in IPF patient lungs. (**A**) H and E staining was performed to reveal the pathological changes in the lungs of IPF patients and non-IPF patients (magnification 200×, bar = 100 μm). (**B**) Masson trichrome staining of collagen deposition in lung tissue (magnification 200×, bar = 100 μm). (**C**–**E**) Immunohistochemical analysis of FN, α-SMA, and phosphorylated S6 expression in lung tissues (magnification 400×, bar = 50 μm).

Moreover, in IPF lung sections, we observed that the alveolar structure disappeared and that substantial lesions formed, manifesting as myofibroblast proliferation (Figure 5A) and collagen deposition (Figure 5B). Accumulation of massive ECM (FN) and α-SMA, markers of fibrosis, indicated the location of the fibrous focus in fibrotic lesions (Figure 5C,D). Parallel to the findings above, we found phosphorylation of S6 in IPF lesions (Figure 5E), indicating the activation of downstream factors in the mTORC1 pathway.

### 3.5. NEN Prevented BLM-Induced EMT and ECM by Inhibiting the mTORC1 Signalling Pathway in Mice

**Figure 6:**
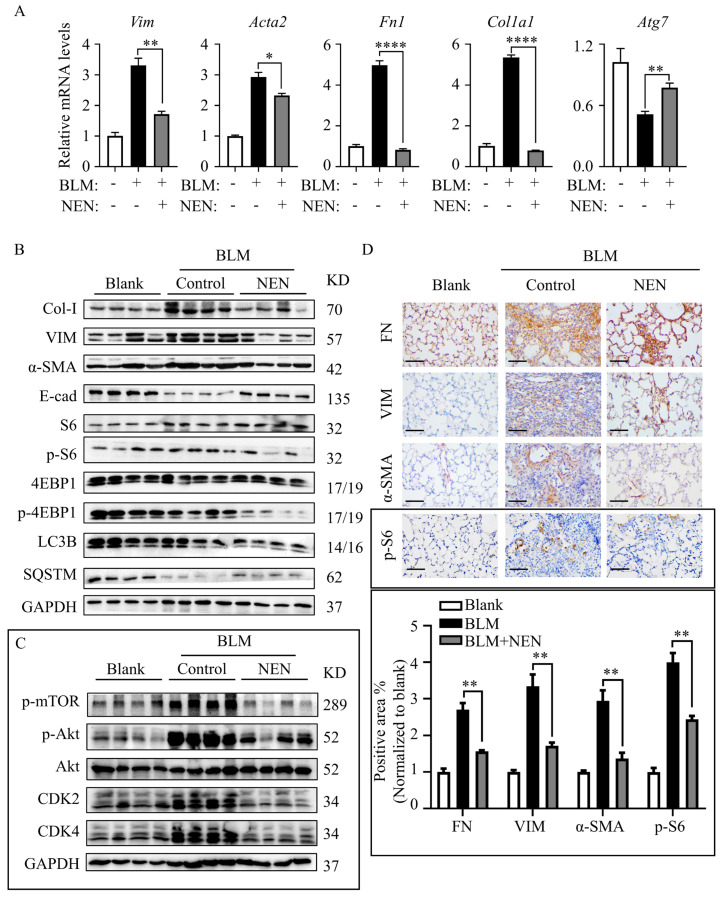
NEN prevented BLM-induced EMT and ECM by inhibiting the mTORC1 signalling pathway in mice. C57BL-6J mice were challenged with BLM or a 0.9% NaCl solution as a vehicle control (blank) at 2.5 mg/kg body weight; the agents were administered with a liquid lung administration nebulizer. Beginning on day 7 post drug (vehicle) delivery, BLM-challenged mice received NEN (20 mg/kg) or vehicle treatment (i.g.) daily for 2 weeks. (**A**) The mRNA levels of *Vim, Acta2, Fn1, Col1a1,* and *Atg7* in mouse lungs were determined by qPCR. The results were normalized to the *Gapdh* level. (**B**) The expression of fibrosis-related proteins, including Col-I, VIM, α-SMA, and E-cad, and mTOR pathway proteins, including S6 and phosphorylated S6 (S235/236), 4EBP1 and phosphorylated 4EBP1 (T70), SQSTM1/p62, and LC3B (I and II), in mouse lung tissues was detected by an immunoblotting assay. (**C**) The mTOR and phosphorylated mTOR (S2448), Akt and phosphorylated Akt (S473), and cell cycle proteins CDK2 and CDK4 in mouse lung tissues were detected by an immunoblotting assay. (**D**) Immunohistochemical staining was performed to analyse the accumulation of FN, VIM α-SMA, and phosphorylated S6 in mouse lungs (magnification 400×, bar = 100 μm). The positive area in lung sections was quantified by ImageJ software, with normalization to the blank control. * *p* < 0.05, ** *p* < 0.01, **** *p* < 0.0001.

**Figure 7:**
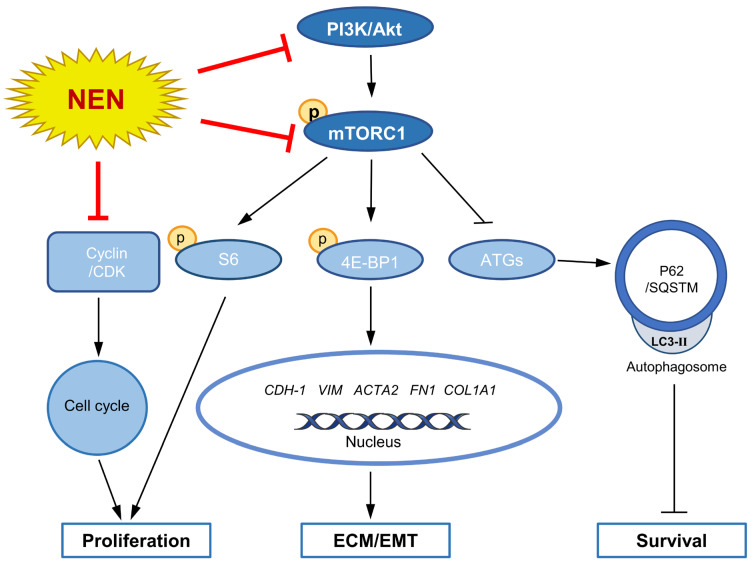
Simplified scheme of the antifibrotic effect of NEN mediated by cell cycle arrest and mTORC1 signalling inhibition.

The in-vitro study revealed mTOR inhibition and autophagy restoration mediated by NEN, so the antifibrotic effect of NEN on BLM-induced IPF mice was evaluated further. The mRNA levels of EMT markers (*Vim*, *Acta2*) and ECM markers (*Fn1*, *Col1a1*) were reduced significantly in the NEN (20 mg/kg) group compared to the BLM group (Figure 6A). Coincidently, the mRNA level of *Atg7* (Figure 6A) was slightly restored by NEN (20 mg/kg) treatment. The protein levels in lysates of whole lung tissues were analysed, and NEN (20 mg/kg) effectively suppressed Col-I and VIM expression and increased E-cad expression (Figure 6B and Supplementary Figure S3A). Furthermore, NEN treatment inhibited the phosphorylation of Akt (S473), mTOR (S2448) (Figure 6C and Supplementary Figure S3D), S6 (S235/236), and 4E-BP1 (S65) and induced SQSTM1/p62 accumulation (Figure 6B and Supplementary Figure S3C). Moreover, NEN blocked the cell cycle in the G1 phase (Figure 6C and Supplementary Figure S3D) by negatively regulating CDK2 and CDK4 in mice. Immunohistochemistry showed that NEN reversed the accumulation of FN, VIM, α-SMA, and phosphorylated S6 in fibrotic foci in lung sections from the BLM group compared with control lung sections (Figure 6D). These data indicated that NEN (20 mg/kg) significantly reduced BLM-induced EMT progression and ECM deposition in vivo, accompanied by PI3K/mTORC1 signalling pathway inhibition and reactivation of noncanonical autophagy (Figure 7).

## 4. Discussion

IPF has an unclear cause and mechanism that involves complex changes in multiple genes and signalling pathways [23]. The development of IPF is accompanied by repeated alveolar epithelial cell damage [24] and excessive fibroblast activation and aggregation, thereby forming scar tissue and fibrotic foci in the lungs and ultimately affecting respiratory function. Alveolar epithelial cells undergoing EMT secrete a range of fibrous growth factors and cytokines, which exacerbate EMT progression and lead to ECM accumulation [25]. These cytokines mainly promote the development of fibrosis and participate in the inflammatory response [26]. TGF-β1 is generally recognized as one of the strongest fibrosis-inducing cytokines [27] and is usually used to induce EMT in alveolar epithelial A549 cells [28]. In our research, TGF-β1-induced A549 cells were employed as an EMT model to explore the mechanism of IPF in vitro. TGF-β1 induced fibrous morphological changes in cells [29], decreased the expression of the epithelial marker E-cad, and increased that of mesenchymal proteins, such as N-cadherin, α-SMA, and VIM [22]. TGF-β1 also has formidable promotive effects on cell proliferation and migration.

NEN was originally developed to eliminate intestinal worms and has recently been studied as a cancer drug [30]. It was reported that preventive administration of niclosamide improved EMT and ECM in pulmonary fibrosis through the Wnt/β-catenin pathway in vitro and in vivo [31], but postdiagnosis therapeutic research is lacking. Studies have found that NEN is a multitarget small-molecule inhibitor in common malignant tumours.

In our research, NEN showed the potential capacity to prevent cell migration and reverse changes in cell morphology in a wound-healing assay performed with either TGF-β1-induced A549 cells or DHLF-IPF cells. NEN slightly increased the expression of the epithelial protein E-cad and decreased that of the α-SMA, VIM, and *CDH2* genes in vitro and in vivo. NEN improved pulmonary function, reduced EMT protein levels and prevented collagen deposition in BLM-induced mice.

mTOR is a Ser/Thr protein kinase that responds to a variety of signals, including growth factors, amino acids, and oxidative stress [32]. Recent studies have shown that activation of the mTORC1 pathway promotes fibrosis, EMT and collagen synthesis [33]. Sftpc-mTOR*^SL1+IT^* transgenic mice, in which mTOR is active, are relatively sensitive to BLM. After exposure to BLM, type 2 alveolar epithelial cells from treated mice exhibit more severe fibrotic changes and lower lung compliance than those from control mice. Hannah V Woodcock et al. showed that inhibiting the mTORC1/4E-BP1 axis played a key role in suppressing TGF-β1-induced COL1A1 transcription and translation in myofibroblasts [34]. When investigating the underlying mechanism involving NEN in IPF in vitro and in vivo, NEN was found to negatively regulate mesenchymal proteins and collagen deposition in TGF-β1-induced A549 cells and DHLF-IPF cells. We demonstrated that NEN inhibited the mTORC1 signalling pathway and activated noncanonical autophagy to inhibit accelerated protein synthesis and disrupted cell proliferation linked with IPF development.

We identified that NEN inhibited the phosphorylation of 4E-BP1 and S6 to regulate the synthesis of EMT and ECM proteins. Pirfenidone, an anti-IPF agent, inhibits the proliferation of primary human intestinal fibroblasts and the production of TGF-β1-induced type I collagen mainly by inhibiting TGF-β1-mediated phosphorylation in the TGF-β1/mTOR/p70S6K signalling pathway [35]. As a new type of mTOR inhibitor [36], niclosamide disturbs the phosphorylation of mTOR and its substrates S6K1 and 4EBP1 in a dose-dependent manner during Epstein–Barr virus cleavage and replication [37]. Therefore, the mTORC1 signalling pathway may be a therapeutic target in IPF.

Autophagy can be activated via mTORC1 downregulation [38]. In our study, NEN increased the LC3II/I ratio, showing autophagy activation mediated by mTORC1 inhibition. Autophagy is a process in which autophagosomes are formed by engulfment of a cell’s own proteins or organelles, and autophagosomes combine with lysosomes to degrade their contents, thereby impacting cell metabolism [39]. Autophagy is related to the biological process of fibrosis but has a dual role in lung fibrosis [40]. On the one hand, Sandra Cabrera et al. first demonstrated the use of *Atg4b*-deficient mice, which exhibit disrupted autophagy, as a model to study the relationship between autophagy and fibrosis in vivo. After BLM treatment, *Atg4b*^−/−^ mice showed increased alveolar and bronchiolar epithelial cell apoptosis, more extensive and severe fibrosis, increased collagen accumulation, and dysregulated expression of ECM-related genes. These findings indicate that autophagy plays a vital role in protecting epithelial cells from inflammation and fibrosis [41]. In addition, rapamycin was found to enhance autophagy, protect alveolar epithelial cells from apoptosis and reduce the inflammatory effect in silica nanoparticle (SiNP)-induced pulmonary fibrosis mice [42]. In contrast, SiO_2_-activated macrophages promoted the proliferation and migration of fibroblasts through MCPIP1/p53-mediated autophagy activation [43].

The noncanonical autophagy pathway is a process that does not function through the canonical autophagy pathway or mediate the entry of intracellular material into lysosomes; instead, it causes the lipidation of LC3. Our results revealed that NEN enriched LC3II and SQSTM1/p62 expression, conforming to noncanonical autophagy. Studies have shown that niclosamide induces canonical autophagy through feedback-mediated downregulation of mTORC1 [44]. However, Haijun Liu et al. found that niclosamide also induced nonstandard LC3 lipidation (NCLL), which is independent of the ULK1 complex and Beclin-1 complex but dependent on the ubiquitin-like binding system [45]. Nintedanib, but not niclosamide, induced noncanonical autophagy during IPF treatment. Sunad Rangarajan found that nintedanib-induced autophagy is ATG7-independent and Beclin-1-dependent and, thus, represents a noncanonical pathway [46]. Moreover, accumulated SQSTM1/p62, as an adaptor between FN1 and the autophagosomal membrane, is closely related to FN1 degradation. Knockdown of SQSTM1/p62 results in excessive FN1 accumulation [47]. Min Li et al. found that niclosamide could not inhibit lysosomal degradation but increased lysosomal permeability, thereby affecting lysosomal H^+^ leakage, which is the same effect as that achieved with an H^+^-ATPase inhibitor. Inhibition of lysosomal function induces autophagy through feedback-mediated downregulation of mTORC1 activity [48]. Our results revealed that Beclin-1-independent noncanonical autophagy enhanced by NEN treatment might help resolve fibrosis development.

In conclusion, we reported that NEN inhibited PI3K-mTORC1 downstream signalling and Beclin-1-independent autophagy, contributing to suppression of EMT and collagen deposition in both TGF-β1-induced epithelial cells and primary human fibroblasts and leading to an improvement in BLM-induced pathological injury and pulmonary function. Our research suggests that NEN is a potential therapeutic agent for IPF.

## Data Availability

All data generated or analysed during this study are included in this published article and are available from the corresponding author upon reasonable request.

## Supplementary Materials

The following supporting information can be downloaded at: https://www.mdpi.com/article/10.3390/cells11030346/s1, Figure S1: Densitometry was performed to quantitatively analyze the indicated protein levels, corresponding to Figure 3E, Figure S2: Densitometry was performed to quantitatively analyze the indicated protein levels, Figure S3: (A)The body weight of each mouse was monitored and recorded daily. Quantitative analyses of protein expression by an immunoblotting assay, corresponding to Figure 6B. Densitometry of fibrosis-related proteins (B) and mTORC1 pathway proteins (C), with normalization to the GAPDH level. Densitometry was performed to quantitatively analyze the indicated protein levels, corresponding to Figure 3E (D), with normalization to the GAPDH level. *** *p* < 0.001, **** *p* < 0.0001.

## Abbreviations

NEN	niclosamide ethanolamine salt.
mTOR	mammalian target of rapamycin.
IPF	idiopathic pulmonary fibrosis.
EMT	epithelial–mesenchymal transition.
ECM	extracellular matrix.
TGF-β1	transforming growth factor-β.
BLM	bleomycin.
DHLF-IPF	diseased human lung fibroblasts, idiopathic pulmonary fibrosis.
FVC	forced vital capacity.
Cdyn	dynamic compliance.
E-cad	E-cadherin.
VIM	vimentin.
α-SMA	alpha-smooth muscle actin.
FN	fibronectin.
Col-I	type I collagen.
