# Supplementary material for: Niclosamide Ethanolamine Salt Alleviates Idiopathic Pulmonary Fibrosis by Modulating the PI3K-mTORC1 Pathway
Source: Cells. 2022 Jan 20;11(3):346. doi: 10.3390/cells11030346 (PMC8834116; doi:10.3390/cells11030346)
Supplement: Supplementary file 1 [file cells-11-00346-s001.zip › cells-1493927-supplementary.pdf]

Supplementary

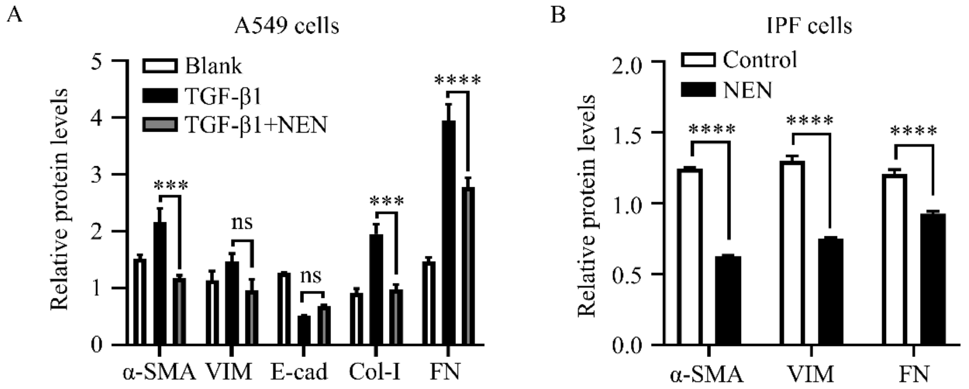

Figure S1. Densitometry was performed to quantitatively analyze the indicated protein levels, corresponding to Figure 3E **(A)** and Figure 3G **(B)**. Normalized to the GAPDH level.

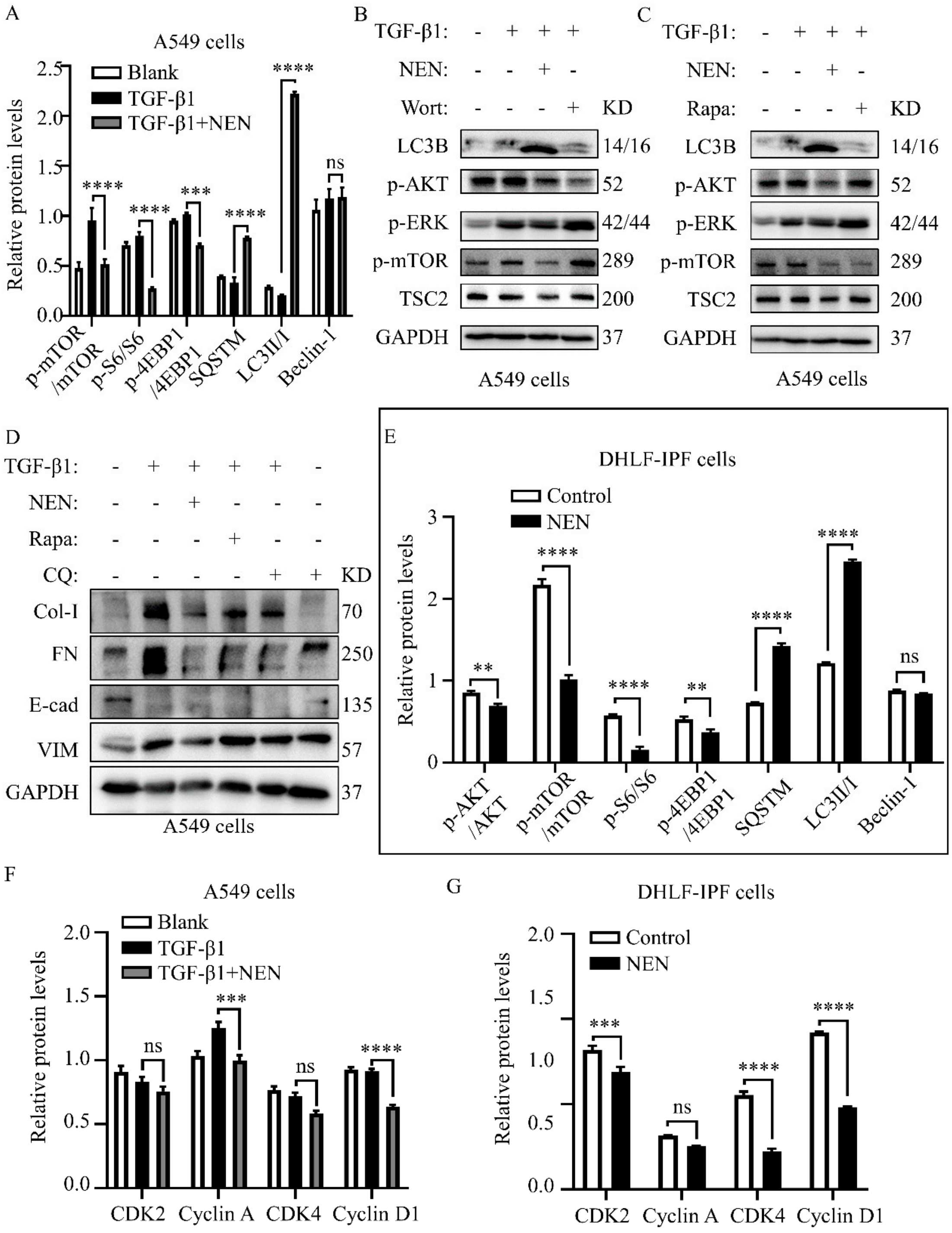

Figure S2. Densitometry was performed to quantitatively analyze the indicated protein levels, corresponding to Figure 4B **(A)**, Figure 4E **(E)**, Figure 4G **(F)**, and Figure 4I **(G)**. Normalized to the GAPDH level. **(B-D)**: Control (blank) or TGF-β1-induced A549 cells were treated with vehicle, NEN (0.5 μM), Rapa (rapamycin) (20 nM), CQ (5 μM) or Wort (wortmannin) (1 μM) for 24 h. Protein expression was detected by an immunoblotting assay. GAPDH was used as an internal reference. $^{**}P<0.01$, $^{****}P<0.0001$.

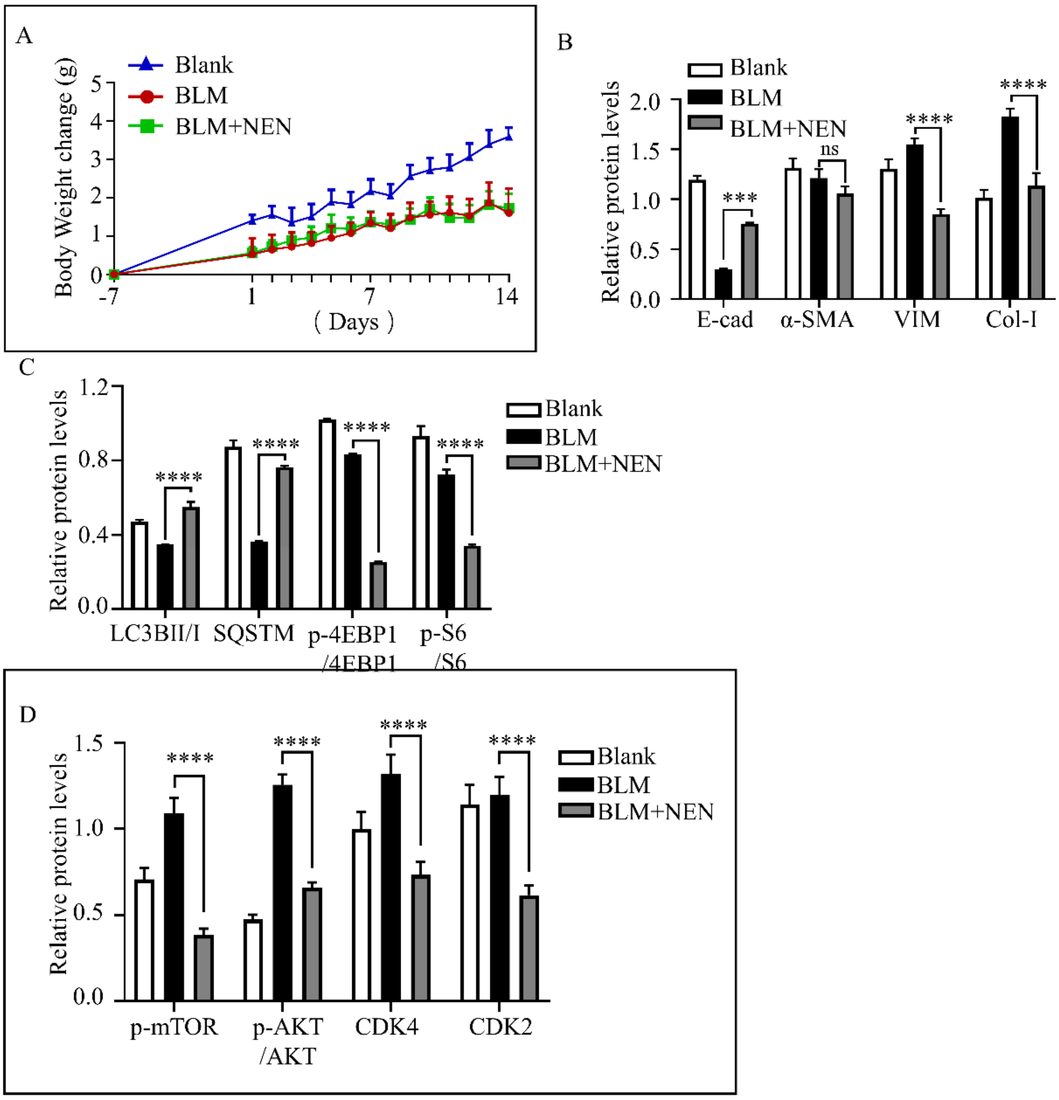

Figure S3. (A) The body weight of each mouse was monitored and recorded daily. Quantitative analyses of protein expression by an immunoblotting assay, corresponding to Figure 6B. Densitometry of fibrosis-related proteins **(B)** and mTORC1 pathway proteins **(C)**, with normalization to the GAPDH level. Densitometry was performed to quantitatively analyze the indicated protein levels, corresponding to Figure 3E **(D),** with normalization to the GAPDH level. ***P<0.001, ****P<0.0001.
